# Intersectional relationships between age, sex, ethnicity, nationality and experience of racism in the UK using different ethnicity categorisations: A comparative study using survey data

**DOI:** 10.1016/j.jmh.2025.100384

**Published:** 2025-12-11

**Authors:** Joseph Lam, Aaron Koay, Mario Cortina-Borja, Robert Aldridge, Ruth Blackburn, Katie Harron

**Affiliations:** a Great Ormond Street Institute of Child Health, University College London, UK; b Institute of Global Health, University College London, UK; c Institute for Health Metrics and Evaluation, University of Washington, USA

**Keywords:** MAIHDA, Intersectionality, Multilevel models, Health inequality, Racism, Ethnicity, Quantitative methods

## Abstract

Traditionally, research has relied on broad ethnic categories such as "Asian," "Black," "White," "Mixed," and "Other." These categories often mask significant variations in experiences and outcomes among ethnic subgroups. The Intersectional Multilevel Analysis of Individual Heterogeneity and Discriminatory Accuracy (I-MAIHDA) approach has become an increasingly recognised quantitative method to study intersectional health inequities. There is a need for better understanding of how the granularity with which ethnicity is measured impacts the interpretation of I-MAIHDA. We used the Evidence from Equality National Survey: A Survey of Ethnic Minorities During the COVID-19 Pandemic, 2021 (EVENS) study, a cross-sectional survey conducted between February and November 2021, including 14,221 individuals with 21 ethnic categories. We constructed intersectional social strata using sex, age, ethnicity and UK nationality. We compared models using 21-category and 5-category ethnicity on describing predicted lifetime experience of racism. Overall, 65% of participants reported experiencing racism in their lifetime. The 5-category model has a higher interaction effect compared to 21-caterogy model due to artefacts from coarse ethnic categorisation. While the interaction effects in 21-category model are smaller, they are potentially more meaningful. The 21-cateogory models revealed significant variations within coarse ethnic groups, showing that individuals from Black Caribbean, African and mixed backgrounds had a higher likelihood of experiencing racism, regardless of UK nationality. The 5-category model failed to attribute the protective effect of not being UK nationality to lower predicted experience of racism in White other backgrounds. Our study demonstrates that using more granular ethnicity categories can lead to more accurate and specific insights in characterising inequities when applying quantitative intersectional approaches, over and above coarse ethnicity groupings used in I-MAIHDA or traditional non-interactive models.

## Highlights

• Careful categorisation and theorising of what ethnicity means is crucial in estimating intersectional health inequalities.
• There is a need for better understanding of how different approaches of measuring and analysing ethnicity impact the interpretation of intersectional social contexts.
• We compared coarse (5-category) and granular (21-category) ethnicity in their ability to explain variation of experience of racism.
• More granular ethnicity enables better description of intersectional disadvantages. This has important implications for study design and analytical approaches to evaluating ethnic health inequities.

## 1 Introduction

The importance of considering ethnicity in research is being increasingly recognised, not only because of the progression in societal awareness of ethnic disparities in health and social outcomes, but also because ethnic identity may serve as a meaningful proxy for describing social contexts in which these disparities arise (Redwood and Gill, 2013; Williams et al., 2019). Typically, health and social science research uses coarse, higher-level ethnic categories (“Asian”, “Black”, “White”,” Mixed”, “Other”), as this categorisation is often readily available, aligns with categories used in previous research, and maps easily to Census categories, making population comparisons possible. However, such broad groupings are known to mask potentially meaningful variations in health outcomes and social contexts (Lett et al., 2022; Senior and Bhopal, 1994). For example, among individuals classified as “Black” (such as Black Caribbean and African), there is evidence of differential experiences of racism, mental health outcomes, and clinical risk score performances (Hatch et al., 2016; Knowles et al., 2022; Movva et al., 2023). Collapsing multiple categories so that people from minoritised groups are categorised as “Mixed” or “Other” can also lead to insights for these populations being biased or unactionable in analysis and policy (Lett et al., 2022; Huyser et al., 2021).

A previous bibliographical review highlighted a lack of explicit theorising of what ethnicity represents in aggregating, modelling and analytical practice in health research (Lam et al., 2023). Whilst more granular ethnicity data are being increasingly collected in some surveys (Shlomo et al., 2023) and electronic health records (Pineda-Moncusí et al., 2024; Akbari et al., 2022), there remain barriers to using more granular ethnicity characterisations in research. Ethnicity has typically been theorised as an individual-level characteristic that might be related to outcomes, rather than one of many social contexts that operates interdependently to shape patterns of privileges and disadvantages. Of note, in this study, ethnicity is used as a proxy for social contexts shaped by racism, a fundamental cause for health inequity, in conjunction with other societal and systemic pathways of discrimination (Lett et al., 2022; Bécares et al., 2022). Intersectionality is a critical theoretical framework positing that systems of domination – such as racism and sexism – are mutually constitutive in structuring differential patterns of inequities (Crenshaw, 1989; Collective, 1977; Collins, 2022). This means that researchers interested in health equity should move beyond a ‘single-axis’ approach (e.g. studying the influence of ethnicity in isolation) to studying how different social contexts may interact to produce compounded inequity (e.g. the interplay of ethnicity, gender and socioeconomic class). As a novel methodology, intersectional multilevel analysis of individual heterogeneity and discriminatory accuracy (I-MAIHDA, (Evans et al., 2024)) enables researchers to quantify differential inequities in outcomes experiences by individuals situated within different intersectional social contexts using an intercategorical approach (McCall, 2005), i.e. comparing inequities across intersectional social strata constructed strategically using analytical categories. I-MAIHDA has become a prominent approach to study intersectional health inequities, with widespread applications from opioid-related deaths, adolescent smoking, to longitudinal MAIHDA that describes changes in mental health trajectories by social contexts (Persmark et al., 2020; Evans, 2019; Bell et al., 2024). There is a now a need for developing a precise understanding of how different approaches of measuring and analysing ethnicity impact the interpretation of intersectional inequities. We therefore aimed to demonstrate the additional insights gained from i) using more granular ethnicity categorisations by comparing analyses using 5- and 21-category ethnicity data; and ii) using an analytical approach that considers ethnicity as part of an intersectional social context in which inequities operate. Specifically, we use data from a cross-sectional survey that collected information on ethnicity and experiences of racism during the COVID-19 pandemic. We use I- MAIHDA to examine whether modelling the interactions between social contexts explains people’s experiences of racism better than considering individual characteristics additively. Additionally, we evaluate whether there are particular social strata where people disproportionately experienced racism, which would have been missed if coarse ethnic categories were used.

## 2 Methods

### 2.1 Data

We used the Evidence from Equality National Survey: A Survey of Ethnic Minorities During the COVID-19 Pandemic, 2021 (EVENS) study, a cross-sectional survey conducted between February and November 2021 (Shlomo et al., 2023). The EVENS dataset was accessed via the UK Data Service (Project ID: 254,768). The dataset contains 14,221 individuals from 21 ethnic categories. Ethnicity was conceptualised and measured as a culturally and socially constructed identity. Detailed sampling approaches are described in Shlomo et al. (2023). After excluding individuals who did not wish to report whether they were UK nationals (*n* *=* 172), the study analysed data from 14,043 individuals. We used unweighted data for this study.

### 2.2 Ethical considerations

EVENS data have been de-identified and are accessible to UK Data Service registered users for non-commercial uses (project id: 254,768). EVENS was approved by The University of Manchester’s Research Ethics Committee (UREC 3, Ref 2021–10,455–17,768).

### 2.3 Outcome

EVENS (Ellingworth et al., 2023) explicitly asked about experiences of racism at different settings, modalities and timing of exposure, such as racist physical assault (34.5 % participants reported), discrimination in house seeking (18.7 %), employment (29.4 %), and other social settings such as in the public (29.9 %). For this study, we have clustered all experiences of racism to a binary “any lifetime experience of racism”.

### 2.4 Intersectional social strata

We used self-identified and self-reported sex (male/female), age at interview (18–30, 31–45, 46–59, 60–74, 75+ years), ethnicity and UK nationality status to construct the intersectional social strata. The selection of these intersectional axes are informed by previous research (Michelle Gyimah et al., 2022; Scobie et al., 2020; Shannon et al., 2022). A great majority (>70 %) of EVENS participants are born in the UK, with an average age of 42. Non-UK nationality is used as a proxy measure for migrant status and a shorter time living in the UK. We expected more younger males and females from minoritised ethnic backgrounds to have experienced racism in their lifetime. We expect non-UK nationals, as a proxy measure for being migrants, to have experienced more racism in their lifetime compared to their UK national counterparts in similar social strata.

Ethnicity was self-identified and self-reported via an online data collection platform, except for people from the Roma and Gypsy Traveller community (*n* *=* 324), where a community interviewer led face-to-face interviews to record responses. Participants were asked to select from a list of ethnic categories (Table 1) and were prompted to complete a free-text self-description of their ethnic identity. This study only included the categorical ethnicity responses. To examine the impact of using coarse ethnic categories, we clustered the responses into 5 categories (White, Black, Asian, Mixed, Other).

**Table 1:** Descriptive statistics of individual observations (*N* = 14,043) in the study data. participants who responded with “prefer not to say” for the question, “are you a UK national? (*n* = 172” were excluded from this study.

			*N*	%
Total			14,043	100
Sex				
		Female	7905	56.3
		Male	6138	43.7
Ethnicity				
	Asian		3833	27.3
		Asian: Bangladeshi	400	2.9
		Asian: Chinese	648	4.6
		Asian: Indian	1274	9.1
		Asian: Pakistani	853	6.1
		Asian: Any other Asian background	658	4.7
	Black		1756	12.5
		Black: African	1023	7.3
		Black: Caribbean	562	4.0
		Black: Any other Black/African/Caribbean background	171	1.22
	Mixed		1394	9.9
		Mixed: White and Asian	513	3.7
		Mixed: White and Black African	158	1.1
		Mixed: White and Black Caribbean	352	2.5
		Mixed: Any other mixed/multiple background	371	2.6
	White		5973	42.5
		White: Eastern European	356	2.5
		White: English / Welsh / Scottish / Northern Irish / White British	4506	32.1
		White: Gypsy/Traveller	251	1.8
		White: Irish	113	0.8
		White: Roma	73	0.5
		White: Any other White background	674	4.8
	Other		1087	7.7
		Other: Arab	149	1.1
		Other: Any other ethnic group	266	1.9
		Jewish	672	4.8
Age Category				
		18 - 30 years	4416	31.5
		31 - 45 years	4687	33.4
		46 - 59 years	2718	19.4
		60 - 74 years	1703	12.1
		75+ years	519	3.7
UK Citizen				
		Yes	11,971	85.3
		No	2072	14.8
Experienced Racism in Lifetime				
		No	4932	35.1
		Yes	9111	64.9

Out of 420 possible combinations (sex(2)*age(5)*ethnicity(21)*UK nationality(2)), we constructed 326 intersectional social strata for 21-category ethnicity; out of 100 possible combinations(sex(2)*age(5)*ethnicity(5)*UK nationality(2)), we constructed 92 intersectional social strata for 5-category ethnicity. The rest of the combinations were empty and hence not created.

### 2.5 Statistical analysis

#### 2.5.1 Quantitative representation of intersectionality: intersectional multilevel analysis of individual heterogeneity and discriminatory accuracy

I-MAIHDA is a multi-level, descriptive approach that treats socio-demographics (such as ethnicity and age) as social contexts in which people live, instead of individual characteristics (C. R. Evans et al., 2024). Using this approach, study populations are grouped into social strata defined by key axes of marginalization and inequities, such as sex, ethnicity and disability. I-MAIHDA then estimates (a) the probability of experiencing the outcome in each stratum, and (b) whether there are multiplicative intersectional interactions beyond the independent additive effects of each of the socio-demographic variables (in other words, whether there are between-stratum differential effects on the probability of experiencing the outcome even when those variables are accounted for). Detailed methods for I-MAIHDA are described elsewhere (C. R. Evans et al., 2024).

#### 2.5.2 Multilevel logistic regression models

Cross-sectional I-MAIHDA analysis has a two-level hierarchical structure, with social strata at level 2, and individuals at level 1. We estimated the predicted probability of experiencing racism in each stratum using two random-intercept multilevel logistic regression models.

Model 1: Null Intersectional Model

Model 1 did not include any covariates and had a random intercept on social strata. This can be written as

(1) $$\text{logit}\left(\pi j\right)\equiv \log\left(\frac{\pi j}{1-\pi j}\right)=\beta 0+\text{uj}$$

where πj denotes the probability of experiencing racism for individuals in stratum j, β0 denotes the intercept, and uj denotes the stratum-specific random effect.

Model 2: Additive Main Effects Model

Model 2 included all socio-demographic variables (sex, age, ethnicity, UK nationality) as covariates with fixed-effect regression coefficients, i.e. additive main effects. The random intercept of social strata explains the remaining between-stratum variance. This can be written as

(2) $$\text{logit}\left(\pi j\right)\equiv \log\left(\frac{\pi j}{1-\pi j}\right)=\beta 0+\beta 1\;\chi 1j+\ldots +\beta p\;\chi \text{pj}+\;\text{uj}$$

For model 1 and 2, we calculated the Variance Partition Coefficient (VPC), which describes the proportion of variance found between strata. The VPC is calculated by: between-stratum variance / (between-stratum variance + 3.29 (within-stratum-between-individual variance)). In model 2, VPC is interpreted as the proportion of between-stratum variance remaining after adjusting for additive effects, hence attributable to interaction effects. We expect the VPC to reduce substantially in model 2. Merlo (2018) proposed a framework for interpreting VPC of 0–1, 1–5, 5–10, 10–20, 20–30, and above 30, as absent, very small, small, less large, fairly large and very large respectively. These cut-offs are intended as heuristics to aid interpretation rather than as absolute thresholds (Merlo et al., 2019).

We can quantify the extent to which between-stratum variance is reduced by calculating the proportional change in variance (PCV) between models. A higher PCV suggests that a high proportion of between-stratum variance is accounted for by the contributions of additive main effects.

We compared PCV for models with 21-category ethnicity (model 1A and model 2A) and 5-category ethnicity (model 1B and model 2B). We approximated 95 % confidence intervals for each model where each sample was re-sampled 1000 times, assuming no sampling joint variability between the fixed and random effects. We conducted robustness checks for the analysis using Markov chain Monte Carlo simulations with Stata package runmlwin (Leckie and Charlton, 2013), which produced largely similar results (supplementary Table 5).

All analysis was conducted using Stata 18 (*StataCorp*, 2023). The code was developed based on the tutorial by C. R. Evans et al. (2024) and is published on UCL Data Repository, 10.5522/04/26,437,789 (Lam, 2024).

## 3 Results

**Fig. 1:**
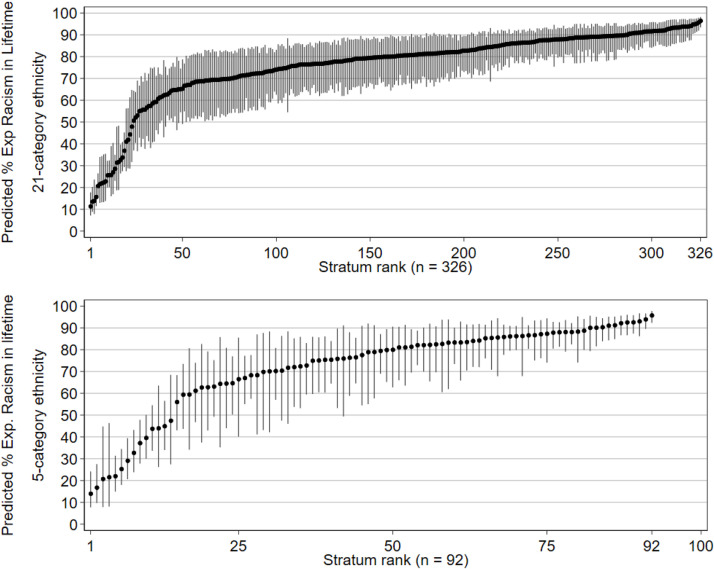
Predicted percentage of people experiencing racism, by stratum (age, sex, ethnicity, UK nationality). Spikes indicate 95 % CIs. Top: predicted percent experiencing racism in lifetime, for all 326 strata using the 21-category ethnicity data, ranked low to high (logistic model 1B). bottom: predicted percent experiencing racism in lifetime, for all 92 strata using 5-category ethnicity data, ranked low to high (logistic model 2B).

Overall, 65 % of participants reported any experience of racism in their lifetime (Table 1). There was a large between-stratum difference in lifetime experience of racism, with the percentage of people within a stratum reporting an experience of racism ranging from ∼10 % to >90 % (Fig. 1).

### 3.1 21-category vs 5-category ethnicity strata

Social strata constructed using 5-category ethnicity generally had larger sample sizes, with 79.3 % strata having more than 10 participants, compared to 55.8 % in strata using 21-category ethnicity (Appendix Table A.1).

**Fig. 2:**
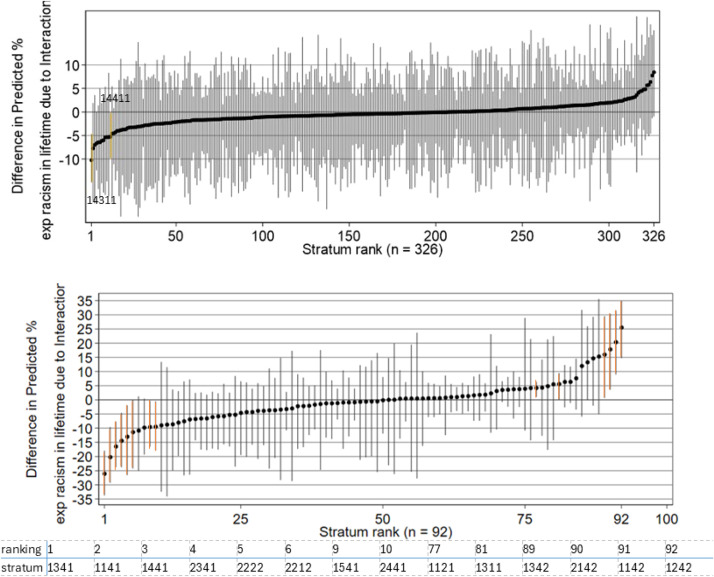
Difference in predicted percent experiencing racism in lifetime (beyond additive effect for each stratum), ranked low to high. spikes indicate approximate 95 % CIs. Fig. 2(a), top, strata using 21-category ethnicity. Fig. 2(b), bottom, strata using 5-category ethnicity.

The intercepts in the models are interpreted as the average between-stratum predicted odds of experiencing racism in a lifetime (Table 2). A VPC of 25.9 % describes a fairly large between-stratum inequities in people’s experience of racism. After accounting for additive effects (model 1B, Fig. 2a), VPC reduced to 2.4 %, with a PCV of 92.8 %. The intersectional interaction effects are marginally better than additive effects in characterising the observed inequities in the experience of racism between strata when using 21-category ethnicity. Nevertheless, it is still important to take the interaction effects into account for more precise estimation.

**Table 2:** Parameter estimates for logistic models of experience of racism in lifetime, null intersectional model (a) vs additive main effect model (b), comparing model using social strata constructed from 21-category ethnicity (left) and 5-category ethnicity (right).

		21-category Ethnicity				5-category Ethnicity			
		Logistic Model 1A		Logistic Model 1B		Logistic Model 2A		Logistic Model 2B	
		OR	95 % CI	OR	95 % CI	OR	95 % CI	OR	95 % CI
**Fixed Effects: Regression Coefficients**									
Intercept (Odds)		4.3	3.7–5.0	0.45	0.37–0.56	3.2	2.5–4.1	1.13	0.75–1.7
Sex									
	Female (Ref)			1.00	-			1.00	-
	Male			1.01	0.88–1.16			1.01	0.77–1.3
Ethnicity									
	Asian: Bangladeshi			15.9	10.3–24.5	Asian		5.90	4.0–8.7
	Asian: Chinese			18.4	12.8–26.6	Black		9.43	6.1–14.5
	Asian: Indian			13.3	9.8–18.0	Mixed		6.90	4.5–10.6
	Asian: Pakistani			14.8	10.5–21.0	White		1.00	-
	Asian: Any other Asian background			16.4	11.5–23.2	Other		4.42	3.0–6.6
	Black: African			20.4	14.5–28.6				
	Black: Caribbean			42.5	26.6–67.8				
	Black: Any other Black/African/Caribbean background			31.1	16.1–60.2				
	Mixed: White and Asian			14.8	10.2–21.5				
	Mixed: White and Black African			30.9	16.0–59.9				
	Mixed: White and Black Caribbean			20.2	12.7–32.1				
	Mixed: Any other mixed/multiple background			14.0	9.4–20.7				
	White: Eastern European			7.6	5.2–11.2				
	White: English / Welsh / Scottish / Northern Ireland/ British (Ref)			1.00	-				
	White: Gypsy/Traveller			19.1	11.9–30.5				
	White: Irish			8.7	5.3–14.2				
	White: Roma			8.8	4.8–16.1				
	White: Any other White background			4.7	3.5–6.4				
	Other: Arab			9.6	5.9–15.5				
	Other: Any other ethnic group			14.0	9.3–21.3				
	Jewish			9.9	7.2–13.7				
Age Category									
	18 - 30 years (Ref)			1.00	-			1.00	-
	31 - 45 years			0.93	0.79–1.10			0.87	0.60–1.24
	46 - 59 years			0.94	0.77–1.16			0.95	0.64–1.40
	60 - 74 years			0.56	0.43–0.71			0.56	0.36–0.87
	75+ years			0.36	0.25–0.52			0.28	0.16–0.50
UK Citizen									
	Yes (Ref)			1.00	-			1.00	-
	No			0.56	0.47–0.66			0.76	0.57–1.02
**Random Effects: Variances**									
Stratum-level		1.15	0.91–1.45	0.08	0.05–0.15	1.17	0.84–1.6	0.27	0.18–0.41
**Summary Statistics**									
Variance Partition Coefficient ( %)		25.9		2.4		26.20		7.55	
Proportional Change in Variance ( %)				92.8				77.0

The null intersectional model using 5-category ethnicity (model 2A, Table 2) also demonstrates a fairly large between-stratum differences of experiencing racism with a VPC of 26.2 %. However, after accounting for additive effects (model 2B, Fig. 2b), the VPC reduced to 7.6 %, with a PCV of 77.0 %. This suggests that interaction terms are necessary to accurately characterise the observed inequities in the experience of racism between strata when using 5-category ethnicity.

Fig. 2 shows the residual interaction effects for each stratum and visualise the extent to which the predicted probability of experiencing racism deviates from additive main effects, using 21-category and 5-category ethnicity. Multilevel logistic models are used to estimate strata-level effects on the pattern of inequalities across populations, and not to examine strata-level residuals for particular strata (Evans et al., 2018). In the additive main effect model using 21-category ethnicity, 2 strata on the caterpillar plot have approximated confidence intervals that do not encompass 0 (Fig. 2a). This suggests a small overall interaction effect that deviates from predicted values using additive effects only in the 21-category model. For example, individuals belonging to the bottom 1 stratum (lowest predicted probability) “Female, White British, over 75 years old, UK national (*n* = 177)”, had a predicted probability of experiencing racism of 11.5 % (approximated 95 % confidence interval 7.4 %−17.4 %), which is lower than predictions based on additive effects only of 12.2 % (9.8 %−15 %) (Table 3). This means the intersectional interactions give rise to protective effects for individuals in this stratum. Individuals belonging to the top 1 stratum “Female, Black Caribbean, 18–30 years old, UK national (*n* = 95)” had a predicted probability of experiencing racism of 96.5 % (approximated 95 % confidence interval 93.3 %−98.1 %), that was higher than expected based on additive effects only of 95.0 % (92.9 %−96.5 %). This means the intersectional interactions better described the compounded detrimental effects for individuals in this stratum. For the additive main effect model using 5-catergory ethnicity, there is clear evidence of the model’s predicted values meaningfully deviating from the prediction from additive effects alone (Fig. 2b). These findings support the conclusion that interaction effect needs to be considered to accurately describe inequities in experiencing lifetime racism in both models, and to a larger extent in models using 5-category ethnicity.

**Table 3:** List of five highest and lowest ranked strata for predicted percentage experiencing racism in lifetime using 21-cat ethnicity (top, model 1B) and 5-cat ethnicity (bottom, model 2B). ** white: English/Welsh/Scottish/Northern Irish/ British. data for all strata for 21-category ethnicity is presented in Appendix Table A.3, and 5-category ethnicity in Appendix Table A.4.

Predicted Percentage Experienced Racism (Model 1B). 21-cat ethnicity							
5 Lowest	Sex	Ethnicity	Age Category	UK National	*n*	Predicted % experienced racism	Predicted % from additive model only
1	Female	White**	75+	Yes	177	11.4 (7.1–17.8)	12.2 (9.8–15.0)
2	Male	White	75+	Yes	228	13.6 (8.8–20.3)	14.2 (11.5–17.3)
3	Male	White	60–74	No	1	14.0 (7.8–23.7)	13.8 (11.7–16.2)
4	Female	White	60–74	Yes	427	15.7 (11.5–21.2)	19.2 (17.2–21.3)
5	Female	White	46–59	Yes	632	20.8 (16.1–26.5)	26.3 (24.2–28.5)
5 Highest							
5	Male	Black Caribbean	18–30	Yes	67	94.8 (90.4–97.2)	95.8 (94.0–97.1)
4	Female	Black Caribbean	46–59	Yes	111	95.0 (90.8–97.3)	94.3 (92.0–96.1)
3	Female	Black Caribbean	31–45	Yes	116	95.3 (91.4–97.4)	94.9 (92.7–96.4)
2	Male	Black Caribbean	46–59	Yes	41	95.8 (91.9–97.8)	95.2 (93.1–96.7)
1	Female	Black Caribbean	18–30	Yes	95	96.5 (93.3–98.1)	95.0 (92.9–96.5)
Predicted Percentage Experienced Racism (Model 2B). 5-cat ethnicity							
5 Lowest	Sex	Ethnicity	Age Category	UK National	n	Predicted % experienced racism	Predicted % from additive model only
1	Female	White	75+	Yes	186	14.0 (7.7–24.2)	16.0 (13.2–19.3)
2	Male	White	75+	Yes	240	16.8 (9.7–27.5)	17.7 (14.7–21.2)
3	Male	White	75+	No	3	20.8 (7.8–44.8)	21.4 (17.5–26.0)
4	Female	White	75+	No	2	21.6 (8.1–46.4)	19.4 (15.8–23.7)
5	Female	White	60–74	Yes	488	22.1 (14.9–31.4)	25.8 (23.5–28.1)
5 Highest							
5	Female	Black	46–59	Yes	195	92.6 (86.6–96.0)	89.0 (87.1–90.7)
4	Male	Black	18–30	Yes	275	92.6 (87.0–95.8)	92.1 (90.7–93.4)
3	Male	Black	46–59	Yes	98	93.1 (86.2–96.7)	90.2 (88.3–91.7)
2	Female	Black	31–45	Yes	309	94.0 (89.5–96.6)	91.2 (89.7–92.5)
1	Female	Black	18–30	Yes	343	95.8 (92.3–97.7)	91.2 (89.6–92.5)

### 3.2 Intersectional comparison of experience of racism

Comparing predicted experience of lifetime racism, 5-category ethnicity model failed to identify that young UK-national males and females who identify as having Black: Other and Mixed White and Black African ethnic background had some of the highest predicted probabilities of experiencing lifetime racism (Appendix Table A.3, A.4). Similarly, 5-category ethnicity model did not capture the elevated predicted probability of lifetime racism for White: Gypsy/Traveller group compared to White British and Any Other White backgrounds.

We observed a protective effect of being a non-UK national in both models (Table 2). We examined the strata of non-UK nationals with the highest and lowest percentage of participants experiencing racism across lifetime (Table 4). By matching all social characteristics apart from being a UK national, we observed that the predicted probability of experiencing racism did not vary across the top five strata, which included predominantly young males and females from Black Caribbean backgrounds. All lowest five strata consisted of middle to older age males and females of White: any other White backgrounds. In these strata, the predicted probability of lifetime racism appears to be lower in non-UK nationals than UK nationals. Using 5-category ethnicity, we observed a similar pattern that the predicted probability of lifetime racism was somewhat lower among non-UK nationals (60–70 %) than UK nationals (63–86 %) (Appendix Table A.2).

**Table 4:** List of five highest and lowest ranked strata, comparisons with matching characteristics apart from not being UK nationals, for predicted percentage experiencing racism in lifetime, using 21-category ethnicity.

Predicted Percentage Experienced Racism in lifetime - comparing UK nationals vs Not UK nationals (Excluding White British)							
5 Lowest	Sex	Ethnicity	Age Category	UK National	n	Predicted % experienced racism	Predicted % from additive model only
1	Male	White: Any other White	75+	No	3	31.5 (18.7–47.9)	33.9 (27.7–40.8)
2	Female	White: Any other White	75+	No	2	31.8 (18.8–48.3)	30.1 (24.4–36.6)
3	Male	White: Any other White	60–74	No	8	41.1 (27.4–56.2)	46.8 (41.6–52.0)
4	Female	White: Any other White	60–74	No	16	41.9 (28.8–56.4)	42.4 (37.4–47.6)
5	Male	White: Any other White	31–45	No	50	52.2 (40.4–63.8)	59.4 (54.9–63.8)
Matching characteristics							
1	Male	White: Any other White	75+	Yes	3	44.4 (28.6–61.3)	47.6 (40.4–54.9)
2	Female	White: Any other White	75+	Yes	3	48.0 (31.7–64.7)	43.2 (36.2–50.6)
3	Male	White: Any other White	60–74	Yes	21	50.7 (37.0–64.3)	60.8 (55.7–65.7)
4	Female	White: Any other White	60–74	Yes	27	55.8 (42.2–68.5)	56.6 (51.3–61.7)
5	Male	White: Any other White	31–45	Yes	40	64.8 (52.6–75.3)	72.1 (68.0–75.8)
Predicted Percentage Experienced Racism in lifetime - comparing UK national vs Not UK nationals							
5 Highest	Sex	Ethnicity	Age Category	UK National	n	Predicted % experienced racism	Predicted % from additive model only
5	Female	Black Caribbean	46–59	No	3	91.0 (83.0–95.5)	90.4 (86.4–93.4)
4	Female	Black Caribbean	18–30	No	6	91.6 (84.2–95.8)	91.6 (88.0–94.2)
3	Male	Black Caribbean	46–59	No	1	91.6 (84.0–95.8)	91.8 (88.2–94.4)
2	Female	Black Caribbean	31–45	No	7	91.8 (84.5–95.8)	91.3 (87.6–94.0)
1	Male	Black Caribbean	18–30	No	1	92.1 (84.8–96.0)	92.8 (89.7–95.1)
Matching characteristics							
5	Female	Black Caribbean	46–59	Yes	111	95.0 (90.8–97.3)	94.3 (92.0–96.1)
4	Female	Black Caribbean	18–30	Yes	95	96.5 (93.3–98.1)	95.0 (92.9–96.5)
3	Male	Black Caribbean	46–59	Yes	41	95.8 (91.9–97.8)	95.2 (93.1–96.7)
2	Female	Black Caribbean	31–45	Yes	116	95.3 (91.4–97.4)	94.9 (91.7–96.4)
1	Male	Black Caribbean	18–30	Yes	67	94.8 (90.4–97.3)	95.8 (94.0–97.1)

## 4 Discussion

Our study demonstrates that using more granular information on ethnicity can lead to additional insights in quantitative intersectional research, over and above those found from the standard 5-category ethnicity groupings that are often used in research. In our study, intersectional interaction effects explain the models better than additive effects only. They also shed light on particular strata whose outcomes differ from one might expect from only considering the additive effects of their socio-demographic characteristics.

We directly compared coarse and granular ethnicity data in terms of their ability to explain variations between and within social strata. High interaction effects were observed when using coarse ethnicity categories compared to more granular categories. The interaction effects observed in the 5-category model are possibly inflated from compensating for the unexplained variability within each broad ethnic category. For example, if individuals from a particular ethnicity subgroup (say, Black Caribbean) were younger than individuals from other Black ethnicities and experienced more racism, that would appear as an ethnicity-age interaction (Black-Caribbean * young) in the 21-category model, but not directly observed in the 5-category model. The more granular 21-category ethnicity model reduces the residual variance, where we observe smaller interaction effects. Contrasting the two models, the interaction effects in the 5-category model, albeit smaller, may be more meaningful qualitatively, as they suggest genuine intersectional effects rather than artifacts of omitted dimensions when using coarser ethnicity categories. They also provide insight that cannot be obtained from additive-only models. This result adds to the literature supporting the use of more granular ethnicity to capture nuanced categorical differences and interactions (Scobie et al., 2020; Lam et al., 2023; Movva et al., 2023).

Our findings highlight sizable inequities in experiencing racism over lifetime, in particular for young males and females identifying as from Black Caribbean, Black African (and mixed) backgrounds, regardless of their UK nationality. We noticed an over-representation of younger males and females experiencing racism. This could be potentially attributed to improved recognition and reporting of race-based discrimination over time in the UK, due to initiatives such as zero tolerance to racism, and governmental Commission on Race and Ethnic Disparity (Inclusive Britain, 2022); or that younger generations are simply more likely to have experienced racism compared to previous generations. Original analysis by EVENS’ authors (Ellingworth et al., 2023) did not find a single specific type of racism that drove these figures. Furthermore, re-analysing the dataset with respect to age-period-cohort effects (Bell, 2020) may help clarify cohort or period specific effect beyond what we described in this paper. We did not find a sex-based difference in the experience of lifetime racism, which could be partly attributable to our clustering of different types of racial discrimination.

Contrary to previous findings (Castañeda et al., 2015; Fernández-Reino and Cuibus, 2024), we observed a protective effect of not being a UK national to experiencing lifetime racism. This protective effect appears to be driven by non-UK nationals from “White: Other White Backgrounds”, who had a lower predicted probability of having experienced racism, compared to their UK national counterparts. This group could refer to people who lived in other White-majority or ethnically homogenous societies, where racism is less likely to occur to people who identify as white, such as Australia, North America, or certain parts of South Africa. If UK nationality is a valid proxy for individuals’ length of stay in the UK, our findings could suggest that the longer people from Other White Backgrounds live in the UK, the higher the probability that they will have experienced racism in their lifetime.

The limitation of using UK nationality as a proxy measure for migrant status and shorter time stayed in the UK can be directly addressed. Country of origin and time in the UK data are available with EVENS for some of the participants, for example, 1795 non-UK nationals stayed in the UK for a median of 5 years (Interquartile range: 2–11), compared to most UK nationals who stayed in the UK their entire life (median age = 40, interquartile range: 28–54). Future studies using EVENS could further investigate if time in the UK mediates the relationship between UK nationality and experience of racism. It is pertinent to note that this migrant-protective effect did not extend to all ethnic groups, where over 90 % of young, mixed Black African and Black Caribbean males and females were predicted to have experienced racism in their lifetime, regardless of their UK nationality. Additionally, the overall protective effect of UK nationality was attenuated in 5-category ethnicity models, and the specific findings for “White: Other White Backgrounds” were largely concealed.

There are some limitations concerning the measurement of racism in this study. First, the clustering of different types of self-reported racism (e.g. physical assaults, house-seeking and employment) into a binary measure of “any lifetime experience of racism” may obscure nuances in the different modalities of racism experienced by each intersectional stratum. Second, while the participants of the EVENS study were asked to self-report experiences of racism, the extent to which people could, in fact, attribute experiences of discrimination to different grounds, such as racism, remains contested (Scheim and Bauer, 2019). This bears relevance, particularly since we are examining intersectional strata that might be concomitantly exposed to multiple intersecting forms of discrimination (e.g. racism, ageism, sexism and xenophobia). Third, this is an intercategorical intersectional research study seeking to compare experiences of racism across social strata situated at different intersections of social contexts. Nonetheless, the concept of racism is unlikely to be a stable construct across different intersectional strata, i.e. do the questions about experiences of racism hold the same meaning for young Asian males of non-UK nationality, middle-aged Black females of UK nationality and older White Gypsy females of UK nationality? This poses questions about measure validity, which is a challenge in intercategorical intersectional research (Scheim and Bauer, 2019). Recognising these limitations, however, we were still able to demonstrate the value of operationalising and measuring ethnicity as a more granular category to reveal nuanced differences in outcomes.

Our study invites further deliberation on a key theoretical and practical question for researchers studying ethnicity and intersectional effects – are more disaggregated ethnicity categories always better? Equivalently, are finer-measured strata always better? This question is essentially asking: do processes of (ethnic) inequities operate at coarser and/or finer levels of measurement or strata definition? This is not just a technical question – it is one that relates to (in)visibility of certain groups and the social inequities they experience. In our study of experience of racism, it is reasonable to hypothesize and examine, a generalised minoritisation effect for all non-White British groups at level 2 (White British vs any other), followed by more granular ethnicities (say, White Other, Black, Asian, Mixed) at level 1 to capture further variations within non-White British groups. We could also examine whether using a very coarse binary ethnicity variable would have worked better in contextualising individuals’ lives, when they are used to define the strata along with origin and age of migration, region of residence and other variables such as English proficiency. I-MAIHDA invites explicit theorising and gives the capability to flexibly apply different choices of aggregation most appropriate to one’s theory and analysis, which we argue is crucial for modelling ethnic inequities. If the selected level of aggregation is too coarse, intersectional effects may be inflated due to unaccounted ethnic variations (similar to our 5-category model). Using a more granular aggregation, the intersectional effects may be reduced but we are likely to generate more accurate predictions for each stratum. However, if the aggregation is too granular, the strata size would likely be small, which can affect the confidence of estimation, despite partially overcome by shrinkage effects of I-MAIHDA. Researchers need to be forthcoming with the ever challenge and art of balancing variable choices and levels of aggregation, to ensure findings are theoretically sound, interpretable and actionable. In particular, researchers should resist the impression that applying the same ethnic categories used by existing researchers would automatically make the choice appropriate for their research question, or directly comparable to the wider literature (Lam et al., 2023).

## 5 Conclusion

Our study presents a case for the mindful use of granular ethnicity categories in quantitative intersectional research in the statistical, theoretical and practical sense. Improved data on ethnicity is essential to appropriately allocate social and health resources to address ethnic health inequities. I-MAIHDA is an approach which can be readily implemented and provides additional insights into intersectional inequities. Meaningful interpretation of ethnicity within the wider social context in which people live is foundational to tackling health and social inequities.

## Funding sources

JL is funded by the Wellcome Trust [212,953/Z/18/Z]. AK is funded by the UCL-Birkbeck Medical Research Council Doctoral Training Partnership (MR/W006774/1) and UCL International Scholar Award for Doctoral Training.

## Data availability

The authors do not have permission to share data. Data can be accessed via UK Data Service.

## CRediT authorship contribution statement

**Joseph Lam:** Conceptualization, Data curation, Formal analysis, Methodology, Visualization, Writing – original draft, Writing – review & editing, Software. **Aaron Koay:** Methodology, Writing – original draft, Writing – review & editing. **Mario Cortina-Borja:** Methodology, Supervision, Writing – review & editing, Visualization. **Robert Aldridge:** Methodology, Supervision, Writing – review & editing, Visualization. **Ruth Blackburn:** Methodology, Supervision, Writing – review & editing, Visualization. **Katie Harron:** Funding acquisition, Methodology, Supervision, Writing – review & editing, Visualization.

## Declaration of competing interest

The authors declare that they have no known competing financial interests or personal relationships that could have appeared to influence the work reported in this paper.

## Appendix Supplementary materials

Image, application 1
