# Supplementary material for: Intersectional relationships between age, sex, ethnicity, nationality and experience of racism in the UK using different ethnicity categorisations: A comparative study using survey data
Source: J Migr Health. 2025 Dec 11;13:100384. doi: 10.1016/j.jmh.2025.100384 (PMC12765057; doi:10.1016/j.jmh.2025.100384)
Supplement: Supplementary file 1 [file mmc1.docx]

## Appendix A: Supplementary Tables

Table A.1. Sample size of per intersectional social strata, defined by respondent sex, ethnicity, age category and UK citizenship (*n* = 326), compared with ethnicity with 5 categories (*n* = 92).

| Sample Size per Stratum | 21-category Ethnicity | | 5-category Ethnicity | |
| --- | --- | --- | --- | --- |
|  | Number of Strata | % | Number of Strata | % |
| Total number of Strata | 326 | - | 92 | - |
| 100 or More | 34 | 10.4% | 37 | 40.2% |
| 50 or More | 76 | 23.3% | 52 | 56.5% |
| 30 or More | 111 | 34.1% | 59 | 64.1% |
| 20 or More | 135 | 41.4% | 69 | 75.0% |
| 10 or More | 182 | 55.8% | 73 | 79.3% |
| Less than 10 | 144 | 44.2% | 19 | 20.7% |

Table A.2. Comparing Strata with the lowest predicted percent experiencing lifetime racism against their UK national counterpart, using 5-category ethnicity.

| Non-White, Lowest 5. | Sex | Ethnicity | Age Category | UK National | *n* | Predicted % experienced racism | Predicted % from additive model only |
| --- | --- | --- | --- | --- | --- | --- | --- |
| 1 | Female | Other | 60-74 | No | 4 | 59.5 (34.1-80.7) | 73.6 (69.3-77.4) |
| 2 | Female | Mixed | 75+ | No | 1 | 64.4 (35.2-85.8) | 69.9 (63.1-75.8) |
| 3 | Female | Asian | 60-74 | No | 2 | 66.5 (40.1-85.5) | 78.7 (75.3-81.8) |
| 4 | Male | Other | 60-74 | No | 1 | 69.9 (43.1-87.6) | 75.8 (71.8-79.5) |
| 5 | Male | Asian | 31-45 | No | 96 | 70.3 (57.0-80.8) | 89.3 (87.7-90.7) |
| Matching |  |  |  |  |  |  |  |
| 1 | Female | Other | 60-74 | Yes | 125 | 63.2(49.2-75.2) | 68.7 (64.8-72.3) |
| 2 | Female | Mixed | 75+ | Yes | 1 | 70.2 (42.2-88.4) | 64.7 (58.2-70.7) |
| 3 | Female | Asian | 60-74 | Yes | 45 | 79.9 (65.2-89.4) | 74.5 (71.5-77.3) |
| 4 | Male | Other | 60-74 | Yes | 109 | 72.8 (59.4-83.1) | 71.3 (67.6-74.7) |
| 5 | Male | Asian | 31-45 | Yes | 527 | 86.2 (79.5-90.9) | 86.8 (85.3-88.1) |

Table A.3. Full result, predicted percentage of experiencing lifetime racism, using 21-category ethnicity. ^ White: English / Welsh / Scottish / Northern Irish/ British

| sex | ethnicity | age_cat | UK Nationality | n | Predicted % racism | low | high | rank |
| --- | --- | --- | --- | --- | --- | --- | --- | --- |
| Female | Black: Caribbean | 18-30 | Yes | 95 | 96.5 | 93.3 | 98.1 | 326 |
| Male | Black: Caribbean | 46-59 | Yes | 41 | 95.8 | 91.9 | 97.8 | 325 |
| Female | Black: Caribbean | 31-45 | Yes | 116 | 95.3 | 91.4 | 97.4 | 324 |
| Female | Black: Caribbean | 46-59 | Yes | 111 | 95.0 | 90.8 | 97.3 | 323 |
| Male | Black: Caribbean | 18-30 | Yes | 67 | 94.8 | 90.4 | 97.3 | 322 |
| Male | Black: Any other Black/African/Caribbean background | 18-30 | Yes | 29 | 94.2 | 87.5 | 97.4 | 321 |
| Female | Black: Any other Black/African/Caribbean background | 31-45 | Yes | 39 | 94.0 | 87.4 | 97.3 | 320 |
| Male | Black: Caribbean | 31-45 | Yes | 65 | 93.8 | 88.7 | 96.7 | 319 |
| Female | Black: Any other Black/African/Caribbean background | 18-30 | Yes | 34 | 93.8 | 87.0 | 97.2 | 318 |
| Male | Mixed: White and Black African | 31-45 | Yes | 14 | 93.8 | 86.6 | 97.2 | 317 |
| Male | Black: Any other Black/African/Caribbean background | 46-59 | Yes | 8 | 93.7 | 86.4 | 97.2 | 316 |
| Female | Mixed: White and Black African | 18-30 | Yes | 41 | 93.6 | 86.5 | 97.0 | 315 |
| Female | Black: African | 18-30 | Yes | 214 | 93.4 | 89.6 | 95.8 | 314 |
| Female | Mixed: White and Black African | 31-45 | Yes | 32 | 93.4 | 86.0 | 97.0 | 313 |
| Female | Mixed: White and Black African | 46-59 | Yes | 14 | 93.3 | 85.7 | 97.0 | 312 |
| Male | Mixed: White and Black African | 18-30 | Yes | 29 | 93.3 | 85.8 | 96.9 | 311 |
| Male | Mixed: White and Black African | 46-59 | Yes | 2 | 93.0 | 84.8 | 96.9 | 310 |
| Female | Black: Any other Black/African/Caribbean background | 46-59 | Yes | 26 | 92.8 | 84.9 | 96.7 | 309 |
| Male | Black: Any other Black/African/Caribbean background | 31-45 | Yes | 19 | 92.6 | 84.4 | 96.6 | 308 |
| Male | Black: Caribbean | 18-30 | No | 1 | 92.1 | 84.8 | 96.0 | 307 |
| Male | Black: Caribbean | 60-74 | Yes | 12 | 92.0 | 84.9 | 95.9 | 306 |
| Male | Mixed: White and Black Caribbean | 31-45 | Yes | 45 | 92.0 | 85.5 | 95.7 | 305 |
| Female | White: Gypsy/Traveller | 31-45 | Yes | 43 | 92.0 | 85.4 | 95.7 | 304 |
| Male | Black: African | 31-45 | Yes | 101 | 91.8 | 86.9 | 95.0 | 303 |
| Female | Black: Caribbean | 60-74 | Yes | 34 | 91.8 | 84.9 | 95.7 | 302 |
| Female | Black: Caribbean | 31-45 | No | 7 | 91.8 | 84.5 | 95.8 | 301 |
| Male | Black: Caribbean | 46-59 | No | 1 | 91.6 | 84.0 | 95.8 | 300 |
| Female | Black: Caribbean | 18-30 | No | 6 | 91.6 | 84.2 | 95.7 | 299 |
| Male | Black: African | 18-30 | Yes | 179 | 91.6 | 87.1 | 94.7 | 298 |
| Female | Black: African | 31-45 | Yes | 154 | 91.6 | 86.9 | 94.7 | 297 |
| Female | Asian: Chinese | 18-30 | Yes | 121 | 91.5 | 86.4 | 94.8 | 296 |
| Male | Mixed: White and Black Caribbean | 46-59 | Yes | 16 | 91.3 | 83.8 | 95.5 | 295 |
| Female | Asian: Chinese | 46-59 | Yes | 26 | 91.1 | 84.5 | 95.0 | 294 |
| Female | Black: Caribbean | 46-59 | No | 3 | 91.0 | 83.0 | 95.5 | 293 |
| Male | Asian: Any other Asian background | 18-30 | Yes | 107 | 91.0 | 85.6 | 94.5 | 292 |
| Male | Black: Caribbean | 31-45 | No | 2 | 91.0 | 82.9 | 95.4 | 291 |
| Male | White: Gypsy/Traveller | 31-45 | Yes | 59 | 90.8 | 83.7 | 94.9 | 290 |
| Female | Black: African | 46-59 | Yes | 58 | 90.6 | 84.6 | 94.5 | 289 |
| Male | White: Gypsy/Traveller | 18-30 | Yes | 15 | 90.6 | 82.7 | 95.1 | 288 |
| Female | White: Gypsy/Traveller | 46-59 | Yes | 15 | 90.0 | 81.8 | 94.8 | 287 |
| Female | Mixed: White and Black Caribbean | 18-30 | Yes | 109 | 90.0 | 83.2 | 94.2 | 286 |
| Female | Asian: Chinese | 31-45 | Yes | 115 | 89.9 | 84.0 | 93.7 | 285 |
| Female | Asian: Pakistani | 46-59 | Yes | 49 | 89.7 | 83.0 | 94.0 | 284 |
| Male | Black: African | 46-59 | Yes | 49 | 89.7 | 83.1 | 93.9 | 283 |
| Male | Asian: Pakistani | 46-59 | Yes | 66 | 89.7 | 83.2 | 93.9 | 282 |
| Male | Mixed: White and Black African | 18-30 | No | 4 | 89.7 | 78.7 | 95.3 | 281 |
| Female | Black: Any other Black/African/Caribbean background | 60-74 | Yes | 4 | 89.6 | 78.4 | 95.3 | 280 |
| Male | Mixed: White and Black Caribbean | 18-30 | Yes | 69 | 89.6 | 82.1 | 94.1 | 279 |
| Male | Asian: Chinese | 46-59 | Yes | 20 | 89.6 | 82.0 | 94.1 | 278 |
| Male | Black: Any other Black/African/Caribbean background | 18-30 | No | 1 | 89.5 | 78.2 | 95.3 | 277 |
| Male | Mixed: White and Black African | 60-74 | Yes | 1 | 89.4 | 77.9 | 95.3 | 276 |
| Male | Other: Any other ethnic group | 18-30 | Yes | 23 | 89.4 | 81.5 | 94.1 | 275 |
| Male | Mixed: White and Asian | 18-30 | Yes | 82 | 89.4 | 82.8 | 93.6 | 274 |
| Female | Mixed: White and Black Caribbean | 31-45 | Yes | 64 | 89.3 | 81.6 | 94.0 | 273 |
| Male | Asian: Bangladeshi | 46-59 | Yes | 23 | 89.2 | 81.0 | 94.2 | 272 |
| Female | Black: Any other Black/African/Caribbean background | 31-45 | No | 6 | 89.2 | 77.8 | 95.1 | 271 |
| Female | Mixed: White and Black African | 18-30 | No | 8 | 89.2 | 77.9 | 95.0 | 270 |
| Female | Mixed: White and Asian | 46-59 | Yes | 31 | 89.1 | 81.6 | 93.8 | 269 |
| Female | Mixed: White and Black African | 31-45 | No | 6 | 89.1 | 77.7 | 95.0 | 268 |
| Female | Asian: Indian | 46-59 | Yes | 114 | 89.1 | 83.2 | 93.0 | 267 |
| Male | White: Gypsy/Traveller | 46-59 | Yes | 18 | 89.0 | 80.1 | 94.2 | 266 |
| Female | Asian: Any other Asian background | 18-30 | Yes | 119 | 89.0 | 82.9 | 93.1 | 265 |
| Male | Black: Any other Black/African/Caribbean background | 31-45 | No | 2 | 88.9 | 77.2 | 95.0 | 264 |
| Female | Asian: Any other Asian background | 31-45 | Yes | 71 | 88.9 | 82.3 | 93.2 | 263 |
| Female | Black: Any other Black/African/Caribbean background | 46-59 | No | 1 | 88.9 | 77.0 | 95.0 | 262 |
| Male | Asian: Pakistani | 18-30 | Yes | 143 | 88.8 | 83.0 | 92.8 | 261 |
| Female | Mixed: White and Black African | 60-74 | Yes | 4 | 88.8 | 76.9 | 95.0 | 260 |
| Male | Mixed: White and Black African | 31-45 | No | 1 | 88.8 | 76.9 | 94.9 | 259 |
| Female | Mixed: White and Black Caribbean | 46-59 | Yes | 33 | 88.5 | 79.8 | 93.8 | 258 |
| Female | Asian: Bangladeshi | 18-30 | Yes | 103 | 88.5 | 81.3 | 93.1 | 257 |
| Female | Asian: Any other Asian background | 46-59 | Yes | 28 | 88.4 | 80.7 | 93.3 | 256 |
| Female | Asian: Bangladeshi | 31-45 | Yes | 89 | 88.2 | 80.8 | 93.1 | 255 |
| Male | Asian: Any other Asian background | 31-45 | Yes | 66 | 88.1 | 81.1 | 92.8 | 254 |
| Female | Black: Caribbean | 75+ | Yes | 1 | 88.0 | 77.0 | 94.2 | 253 |
| Male | Asian: Bangladeshi | 31-45 | Yes | 51 | 88.0 | 79.9 | 93.1 | 252 |
| Male | Mixed: Any other mixed/multiple background | 31-45 | Yes | 39 | 88.0 | 80.0 | 93.1 | 251 |
| Male | Other: Any other ethnic group | 31-45 | Yes | 30 | 88.0 | 79.5 | 93.2 | 250 |
| Male | Asian: Chinese | 31-45 | Yes | 85 | 88.0 | 81.0 | 92.6 | 249 |
| Male | Black: Any other Black/African/Caribbean background | 60-74 | Yes | 2 | 87.9 | 75.4 | 94.6 | 248 |
| Male | Asian: Chinese | 18-30 | Yes | 93 | 87.9 | 81.1 | 92.5 | 247 |
| Male | Asian: Bangladeshi | 18-30 | Yes | 96 | 87.7 | 80.1 | 92.6 | 246 |
| Female | Mixed: Any other mixed/multiple background | 18-30 | Yes | 77 | 87.7 | 80.2 | 92.6 | 245 |
| Male | Other: Any other ethnic group | 46-59 | Yes | 9 | 87.6 | 78.3 | 93.3 | 244 |
| Female | Asian: Bangladeshi | 46-59 | Yes | 14 | 87.6 | 78.3 | 93.3 | 243 |
| Female | Other: Any other ethnic group | 31-45 | Yes | 35 | 87.6 | 79.1 | 93.0 | 242 |
| Male | Asian: Indian | 46-59 | Yes | 93 | 87.6 | 81.0 | 92.1 | 241 |
| Male | Asian: Any other Asian background | 46-59 | Yes | 17 | 87.5 | 79.1 | 92.9 | 240 |
| Female | White: Gypsy/Traveller | 18-30 | Yes | 33 | 87.1 | 77.7 | 92.9 | 239 |
| Female | Asian: Indian | 18-30 | Yes | 210 | 87.0 | 81.5 | 91.0 | 238 |
| Female | Mixed: White and Asian | 31-45 | Yes | 73 | 86.7 | 79.1 | 91.9 | 237 |
| Female | Mixed: Any other mixed/multiple background | 31-45 | Yes | 70 | 86.7 | 78.8 | 92.0 | 236 |
| Female | White: Gypsy/Traveller | 60-74 | Yes | 21 | 86.6 | 76.3 | 92.8 | 235 |
| Male | Mixed: White and Asian | 31-45 | Yes | 62 | 86.5 | 78.6 | 91.8 | 234 |
| Female | Mixed: White and Asian | 18-30 | Yes | 145 | 86.5 | 79.7 | 91.3 | 233 |
| Female | Other: Any other ethnic group | 46-59 | Yes | 22 | 86.5 | 77.0 | 92.4 | 232 |
| Female | Mixed: Any other mixed/multiple background | 46-59 | Yes | 29 | 86.4 | 77.3 | 92.2 | 231 |
| Female | Asian: Pakistani | 18-30 | Yes | 207 | 86.3 | 80.2 | 90.8 | 230 |
| Male | Mixed: Any other mixed/multiple background | 46-59 | Yes | 20 | 86.3 | 76.9 | 92.3 | 229 |
| Male | Jewish | 18-30 | Yes | 37 | 86.3 | 78.0 | 91.7 | 228 |
| Female | Other: Any other ethnic group | 18-30 | Yes | 35 | 86.1 | 76.9 | 92.0 | 227 |
| Female | Asian: Pakistani | 31-45 | Yes | 172 | 86.1 | 79.6 | 90.7 | 226 |
| Male | Asian: Indian | 31-45 | Yes | 187 | 86.0 | 80.1 | 90.4 | 225 |
| Male | White: Gypsy/Traveller | 60-74 | Yes | 14 | 85.9 | 75.1 | 92.5 | 224 |
| Male | Asian: Pakistani | 31-45 | Yes | 138 | 85.8 | 79.0 | 90.7 | 223 |
| Male | Mixed: White and Asian | 46-59 | Yes | 17 | 85.7 | 76.1 | 91.9 | 222 |
| Female | Black: African | 60-74 | Yes | 6 | 85.4 | 75.4 | 91.8 | 221 |
| Female | Mixed: White and Black Caribbean | 60-74 | Yes | 6 | 85.3 | 73.9 | 92.3 | 220 |
| Female | Jewish | 18-30 | Yes | 67 | 85.1 | 77.1 | 90.6 | 219 |
| Female | Mixed: White and Black Caribbean | 18-30 | No | 3 | 84.9 | 73.3 | 92.0 | 218 |
| Male | Jewish | 31-45 | Yes | 50 | 84.9 | 76.5 | 90.6 | 217 |
| Female | Black: African | 46-59 | No | 12 | 84.7 | 74.8 | 91.2 | 216 |
| Female | Asian: Chinese | 18-30 | No | 59 | 84.6 | 76.0 | 90.5 | 215 |
| Male | Mixed: White and Black African | 75+ | Yes | 1 | 84.4 | 68.5 | 93.1 | 214 |
| Female | Asian: Chinese | 60-74 | Yes | 7 | 84.4 | 73.6 | 91.3 | 213 |
| Female | Asian: Indian | 31-45 | Yes | 221 | 84.4 | 78.3 | 89.0 | 212 |
| Male | Mixed: White and Black Caribbean | 60-74 | Yes | 4 | 84.1 | 72.0 | 91.7 | 211 |
| Male | Mixed: White and Black Caribbean | 46-59 | No | 1 | 84.0 | 71.5 | 91.7 | 210 |
| Male | Black: African | 60-74 | Yes | 8 | 83.9 | 73.3 | 90.8 | 209 |
| Male | Mixed: White and Black Caribbean | 31-45 | No | 1 | 83.8 | 71.4 | 91.5 | 208 |
| Male | Asian: Indian | 60-74 | Yes | 31 | 83.5 | 74.0 | 90.0 | 207 |
| Female | Black: African | 18-30 | No | 58 | 83.3 | 74.5 | 89.4 | 206 |
| Male | Asian: Chinese | 60-74 | Yes | 5 | 83.1 | 71.7 | 90.6 | 205 |
| Female | White: Gypsy/Traveller | 46-59 | No | 1 | 83.0 | 70.1 | 91.1 | 204 |
| Female | Asian: Any other Asian background | 46-59 | No | 17 | 83.0 | 72.5 | 90.0 | 203 |
| Male | Black: African | 46-59 | No | 6 | 82.9 | 71.9 | 90.1 | 202 |
| Male | Asian: Chinese | 18-30 | No | 49 | 82.8 | 73.4 | 89.4 | 201 |
| Female | Asian: Chinese | 31-45 | No | 37 | 82.7 | 73.0 | 89.5 | 200 |
| Male | Black: African | 18-30 | No | 47 | 82.7 | 73.5 | 89.2 | 199 |
| Female | Other: Arab | 18-30 | Yes | 29 | 82.4 | 70.6 | 90.1 | 198 |
| Male | Asian: Indian | 18-30 | Yes | 190 | 82.2 | 75.5 | 87.4 | 197 |
| Male | Asian: Chinese | 46-59 | No | 4 | 82.2 | 70.5 | 89.9 | 196 |
| Female | Other: Arab | 31-45 | Yes | 16 | 82.2 | 69.9 | 90.2 | 195 |
| Female | Asian: Any other Asian background | 18-30 | No | 58 | 82.1 | 72.9 | 88.6 | 194 |
| Male | Other: Arab | 18-30 | Yes | 21 | 82.1 | 69.9 | 90.0 | 193 |
| Male | White: Gypsy/Traveller | 46-59 | No | 1 | 82.0 | 68.6 | 90.5 | 192 |
| Male | Asian: Any other Asian background | 18-30 | No | 40 | 82.0 | 72.3 | 88.8 | 191 |
| Female | Asian: Any other Asian background | 60-74 | Yes | 8 | 82.0 | 70.5 | 89.7 | 190 |
| Male | Other: Arab | 31-45 | Yes | 19 | 81.9 | 69.5 | 89.9 | 189 |
| Male | Asian: Any other Asian background | 60-74 | Yes | 12 | 81.9 | 70.5 | 89.5 | 188 |
| Male | Other: Arab | 46-59 | Yes | 1 | 81.7 | 68.2 | 90.3 | 187 |
| Male | Asian: Pakistani | 60-74 | Yes | 12 | 81.6 | 70.2 | 89.4 | 186 |
| Male | White: Gypsy/Traveller | 31-45 | No | 5 | 81.5 | 68.4 | 90.0 | 185 |
| Male | White: Roma | 18-30 | Yes | 2 | 81.5 | 66.3 | 90.8 | 184 |
| Female | Mixed: Any other mixed/multiple background | 60-74 | Yes | 10 | 81.4 | 69.3 | 89.5 | 183 |
| Male | White: Gypsy/Traveller | 18-30 | No | 5 | 81.4 | 68.1 | 89.9 | 182 |
| Male | Mixed: Any other mixed/multiple background | 18-30 | No | 9 | 81.4 | 69.5 | 89.3 | 181 |
| Female | White: Roma | 18-30 | Yes | 2 | 81.4 | 66.1 | 90.7 | 180 |
| Female | Mixed: White and Asian | 18-30 | No | 27 | 81.3 | 70.5 | 88.8 | 179 |
| Male | Mixed: Any other mixed/multiple background | 18-30 | Yes | 45 | 81.3 | 70.9 | 88.5 | 178 |
| Female | Asian: Bangladeshi | 60-74 | Yes | 1 | 81.2 | 67.9 | 89.8 | 177 |
| Male | Asian: Any other Asian background | 46-59 | No | 7 | 81.2 | 69.5 | 89.1 | 176 |
| Male | White: Irish | 31-45 | Yes | 10 | 81.0 | 67.6 | 89.7 | 175 |
| Female | Other: Arab | 46-59 | Yes | 9 | 81.0 | 67.6 | 89.7 | 174 |
| Female | Asian: Chinese | 46-59 | No | 9 | 80.8 | 68.9 | 88.9 | 173 |
| Female | White: Roma | 46-59 | Yes | 3 | 80.7 | 65.2 | 90.4 | 172 |
| Female | Asian: Bangladeshi | 18-30 | No | 4 | 80.7 | 67.8 | 89.3 | 171 |
| Male | Jewish | 46-59 | Yes | 61 | 80.7 | 71.3 | 87.5 | 170 |
| Female | White: Gypsy/Traveller | 31-45 | No | 7 | 80.7 | 67.3 | 89.4 | 169 |
| Male | Asian: Bangladeshi | 18-30 | No | 8 | 80.6 | 67.9 | 89.2 | 168 |
| Male | Asian: Bangladeshi | 46-59 | No | 1 | 80.6 | 66.9 | 89.4 | 167 |
| Female | Jewish | 31-45 | Yes | 77 | 80.5 | 71.6 | 87.2 | 166 |
| Male | Asian: Bangladeshi | 60-74 | Yes | 2 | 80.4 | 66.8 | 89.3 | 165 |
| Male | Asian: Chinese | 31-45 | No | 17 | 80.3 | 68.9 | 88.3 | 164 |
| Female | Asian: Bangladeshi | 31-45 | No | 6 | 80.2 | 67.1 | 88.9 | 163 |
| Male | White: Irish | 18-30 | Yes | 2 | 80.1 | 65.9 | 89.3 | 162 |
| Female | White: Roma | 31-45 | Yes | 6 | 80.0 | 64.4 | 89.9 | 161 |
| Male | Mixed: White and Asian | 60-74 | Yes | 5 | 80.0 | 67.3 | 88.6 | 160 |
| Male | White: Irish | 46-59 | Yes | 10 | 80.0 | 66.3 | 89.1 | 159 |
| Female | White: Gypsy/Traveller | 18-30 | No | 5 | 80.0 | 66.2 | 89.1 | 158 |
| Female | Asian: Pakistani | 18-30 | No | 15 | 80.0 | 68.4 | 88.1 | 157 |
| Male | Mixed: White and Asian | 18-30 | No | 14 | 79.9 | 68.0 | 88.1 | 156 |
| Female | Black: African | 31-45 | No | 73 | 79.8 | 70.5 | 86.7 | 155 |
| Male | Asian: Pakistani | 46-59 | No | 2 | 79.7 | 67.0 | 88.4 | 154 |
| Female | Asian: Pakistani | 60-74 | Yes | 4 | 79.7 | 67.0 | 88.3 | 153 |
| Female | White: Irish | 18-30 | Yes | 1 | 79.6 | 65.2 | 89.1 | 152 |
| Male | Asian: Pakistani | 31-45 | No | 7 | 79.6 | 67.4 | 88.0 | 151 |
| Female | Mixed: White and Asian | 31-45 | No | 22 | 79.5 | 67.9 | 87.7 | 150 |
| Male | Mixed: White and Asian | 46-59 | No | 1 | 79.4 | 66.2 | 88.4 | 149 |
| Male | Asian: Bangladeshi | 31-45 | No | 2 | 79.3 | 65.6 | 88.5 | 148 |
| Female | Mixed: Any other mixed/multiple background | 31-45 | No | 29 | 79.3 | 67.6 | 87.5 | 147 |
| Male | Mixed: Any other mixed/multiple background | 60-74 | Yes | 10 | 79.2 | 66.3 | 88.1 | 146 |
| Male | White: Roma | 31-45 | Yes | 2 | 79.2 | 62.9 | 89.5 | 145 |
| Male | White: Eastern European | 18-30 | Yes | 6 | 79.1 | 66.5 | 87.9 | 144 |
| Female | Mixed: White and Asian | 60-74 | Yes | 7 | 79.1 | 66.2 | 88.0 | 143 |
| Female | Other: Any other ethnic group | 46-59 | No | 8 | 79.1 | 65.8 | 88.1 | 142 |
| Male | Other: Any other ethnic group | 31-45 | No | 12 | 78.8 | 66.0 | 87.8 | 141 |
| Male | Mixed: Any other mixed/multiple background | 46-59 | No | 1 | 78.5 | 64.7 | 87.9 | 140 |
| Male | Other: Any other ethnic group | 46-59 | No | 5 | 78.4 | 64.7 | 87.8 | 139 |
| Male | White: Gypsy/Traveller | 75+ | Yes | 5 | 78.3 | 62.7 | 88.6 | 138 |
| Male | Asian: Pakistani | 18-30 | No | 12 | 78.3 | 66.0 | 87.0 | 137 |
| Male | Mixed: White and Black Caribbean | 75+ | Yes | 1 | 78.1 | 61.9 | 88.7 | 136 |
| Female | White: Gypsy/Traveller | 75+ | Yes | 4 | 77.9 | 61.9 | 88.4 | 135 |
| Female | Asian: Indian | 18-30 | No | 62 | 77.9 | 68.2 | 85.2 | 134 |
| Female | Mixed: Any other mixed/multiple background | 46-59 | No | 4 | 77.8 | 64.2 | 87.3 | 133 |
| Male | Black: African | 31-45 | No | 55 | 77.8 | 67.6 | 85.5 | 132 |
| Female | White: Irish | 31-45 | Yes | 12 | 77.8 | 63.4 | 87.6 | 131 |
| Female | Asian: Indian | 46-59 | No | 6 | 77.6 | 65.0 | 86.6 | 130 |
| Male | Asian: Indian | 18-30 | No | 41 | 77.4 | 66.9 | 85.3 | 129 |
| Female | Other: Any other ethnic group | 31-45 | No | 20 | 77.3 | 64.4 | 86.5 | 128 |
| Female | Mixed: White and Asian | 46-59 | No | 3 | 77.1 | 63.3 | 86.8 | 127 |
| Male | Mixed: Any other mixed/multiple background | 31-45 | No | 6 | 77.1 | 63.5 | 86.7 | 126 |
| Male | Other: Any other ethnic group | 18-30 | No | 10 | 76.9 | 63.3 | 86.5 | 125 |
| Female | Asian: Pakistani | 46-59 | No | 2 | 76.8 | 63.1 | 86.5 | 124 |
| Female | White: Irish | 46-59 | Yes | 8 | 76.8 | 61.9 | 87.1 | 123 |
| Female | Mixed: Any other mixed/multiple background | 18-30 | No | 19 | 76.8 | 64.0 | 86.0 | 122 |
| Female | White: Eastern European | 46-59 | Yes | 6 | 76.8 | 63.3 | 86.4 | 121 |
| Female | Jewish | 46-59 | Yes | 86 | 76.7 | 67.0 | 84.2 | 120 |
| Female | White: Eastern European | 31-45 | Yes | 53 | 76.6 | 65.6 | 84.9 | 119 |
| Male | Other: Any other ethnic group | 60-74 | Yes | 9 | 76.5 | 62.4 | 86.5 | 118 |
| Male | White: Eastern European | 31-45 | Yes | 18 | 76.5 | 63.9 | 85.7 | 117 |
| Female | Asian: Indian | 60-74 | Yes | 25 | 76.5 | 64.7 | 85.3 | 116 |
| Female | Asian: Any other Asian background | 31-45 | No | 64 | 76.5 | 66.1 | 84.4 | 115 |
| Male | Mixed: White and Asian | 31-45 | No | 18 | 76.5 | 63.8 | 85.7 | 114 |
| Female | Other: Any other ethnic group | 18-30 | No | 30 | 76.4 | 63.8 | 85.6 | 113 |
| Female | Other: Any other ethnic group | 60-74 | Yes | 13 | 76.3 | 62.4 | 86.2 | 112 |
| Female | Black: African | 60-74 | No | 2 | 76.0 | 61.9 | 86.0 | 111 |
| Male | Black: African | 60-74 | No | 1 | 75.9 | 61.7 | 86.0 | 110 |
| Female | Asian: Pakistani | 31-45 | No | 16 | 75.8 | 63.1 | 85.1 | 109 |
| Female | Asian: Indian | 31-45 | No | 46 | 75.7 | 65.1 | 83.9 | 108 |
| Male | Asian: Indian | 46-59 | No | 7 | 75.4 | 62.3 | 85.0 | 107 |
| Female | Mixed: White and Black African | 75+ | No | 1 | 75.1 | 54.5 | 88.4 | 106 |
| Male | Asian: Any other Asian background | 31-45 | No | 39 | 74.8 | 63.2 | 83.6 | 105 |
| Female | Jewish | 18-30 | No | 5 | 74.8 | 61.1 | 84.8 | 104 |
| Female | Asian: Any other Asian background | 75+ | Yes | 2 | 74.5 | 58.8 | 85.7 | 103 |
| Male | White: Eastern European | 46-59 | Yes | 3 | 74.5 | 60.1 | 85.0 | 102 |
| Male | Asian: Any other Asian background | 75+ | Yes | 1 | 74.4 | 58.5 | 85.6 | 101 |
| Male | Asian: Indian | 31-45 | No | 31 | 74.1 | 62.5 | 83.1 | 100 |
| Male | Jewish | 60-74 | Yes | 98 | 74.0 | 63.9 | 82.0 | 99 |
| Female | White: Eastern European | 18-30 | Yes | 25 | 73.6 | 60.7 | 83.4 | 98 |
| Male | Jewish | 18-30 | No | 1 | 73.4 | 59.0 | 84.1 | 97 |
| Female | Jewish | 46-59 | No | 4 | 73.3 | 59.1 | 83.9 | 96 |
| Female | White: Eastern European | 31-45 | No | 98 | 73.2 | 62.9 | 81.5 | 95 |
| Male | White: Roma | 18-30 | No | 13 | 73.0 | 55.8 | 85.3 | 94 |
| Male | Asian: Pakistani | 75+ | Yes | 5 | 72.6 | 56.8 | 84.3 | 93 |
| Male | Asian: Chinese | 60-74 | No | 1 | 72.4 | 56.9 | 83.9 | 92 |
| Female | White: Eastern European | 18-30 | No | 54 | 72.4 | 60.7 | 81.7 | 91 |
| Female | Other: Arab | 60-74 | Yes | 1 | 72.3 | 55.4 | 84.6 | 90 |
| Male | Other: Any other ethnic group | 75+ | Yes | 3 | 72.3 | 55.3 | 84.7 | 89 |
| Female | Other: Arab | 31-45 | No | 18 | 72.2 | 57.0 | 83.6 | 88 |
| Female | Mixed: White and Asian | 75+ | Yes | 1 | 72.2 | 55.4 | 84.4 | 87 |
| Female | Other: Arab | 18-30 | No | 14 | 71.9 | 56.4 | 83.5 | 86 |
| Male | White: Irish | 18-30 | No | 3 | 71.7 | 54.9 | 84.0 | 85 |
| Female | White: Any other White background | 31-45 | Yes | 60 | 71.6 | 61.0 | 80.3 | 84 |
| Male | Other: Arab | 18-30 | No | 9 | 71.5 | 55.5 | 83.4 | 83 |
| Male | Other: Arab | 60-74 | Yes | 2 | 71.4 | 54.3 | 84.0 | 82 |
| Female | Other: Arab | 46-59 | No | 1 | 71.3 | 54.3 | 83.8 | 81 |
| Female | White: Irish | 18-30 | No | 2 | 71.0 | 54.0 | 83.6 | 80 |
| Male | Jewish | 31-45 | No | 5 | 70.6 | 56.1 | 81.9 | 79 |
| Male | White: Irish | 60-74 | Yes | 14 | 70.5 | 54.3 | 82.7 | 78 |
| Female | Jewish | 31-45 | No | 8 | 70.3 | 56.0 | 81.4 | 77 |
| Male | Mixed: White and Asian | 60-74 | No | 2 | 70.1 | 54.1 | 82.4 | 76 |
| Male | Asian: Indian | 75+ | Yes | 7 | 69.9 | 54.1 | 82.0 | 75 |
| Female | White: Irish | 46-59 | No | 12 | 69.9 | 53.5 | 82.4 | 74 |
| Male | Other: Arab | 46-59 | No | 1 | 69.8 | 52.5 | 82.9 | 73 |
| Male | White: Irish | 46-59 | No | 8 | 69.8 | 53.1 | 82.5 | 72 |
| Male | Asian: Pakistani | 60-74 | No | 1 | 69.7 | 53.7 | 82.0 | 71 |
| Male | Mixed: Any other mixed/multiple background | 75+ | Yes | 1 | 69.6 | 52.1 | 82.8 | 70 |
| Male | White: Any other White background | 18-30 | Yes | 19 | 69.4 | 56.5 | 79.9 | 69 |
| Female | White: Irish | 31-45 | No | 5 | 69.4 | 52.5 | 82.3 | 68 |
| Female | Asian: Pakistani | 75+ | Yes | 2 | 69.4 | 52.6 | 82.3 | 67 |
| Male | White: Roma | 31-45 | No | 4 | 69.4 | 50.7 | 83.3 | 66 |
| Female | Asian: Indian | 75+ | Yes | 3 | 69.3 | 53.1 | 81.9 | 65 |
| Female | White: Roma | 46-59 | No | 10 | 69.2 | 50.9 | 83.0 | 64 |
| Female | White: Roma | 18-30 | No | 14 | 69.1 | 51.2 | 82.7 | 63 |
| Male | White: Roma | 46-59 | No | 6 | 69.0 | 50.4 | 83.1 | 62 |
| Male | White: Eastern European | 18-30 | No | 15 | 68.7 | 54.5 | 80.1 | 61 |
| Female | Mixed: Any other mixed/multiple background | 60-74 | No | 2 | 68.7 | 52.3 | 81.5 | 60 |
| Female | Mixed: White and Asian | 60-74 | No | 3 | 68.7 | 52.6 | 81.3 | 59 |
| Male | White: Irish | 31-45 | No | 3 | 68.6 | 51.4 | 81.9 | 58 |
| Female | Asian: Any other Asian background | 60-74 | No | 2 | 68.6 | 52.8 | 81.0 | 57 |
| Male | Other: Arab | 31-45 | No | 8 | 68.2 | 51.6 | 81.2 | 56 |
| Male | White: Eastern European | 60-74 | Yes | 1 | 68.0 | 51.7 | 80.8 | 55 |
| Female | Other: Any other ethnic group | 60-74 | No | 2 | 67.1 | 50.3 | 80.5 | 54 |
| Female | White: Eastern European | 46-59 | No | 11 | 67.1 | 52.2 | 79.2 | 53 |
| Female | White: Irish | 60-74 | Yes | 10 | 66.9 | 50.0 | 80.4 | 52 |
| Male | White: Any other White background | 46-59 | Yes | 45 | 66.7 | 54.9 | 76.8 | 51 |
| Female | White: Eastern European | 60-74 | Yes | 3 | 65.4 | 49.1 | 78.7 | 50 |
| Female | Jewish | 60-74 | Yes | 111 | 65.2 | 54.5 | 74.5 | 49 |
| Male | White: Eastern European | 46-59 | No | 7 | 65.2 | 49.5 | 78.1 | 48 |
| Female | White: Roma | 31-45 | No | 11 | 64.9 | 46.2 | 79.9 | 47 |
| Male | White: Any other White background | 31-45 | Yes | 40 | 64.8 | 52.6 | 75.3 | 46 |
| Female | Jewish | 75+ | Yes | 28 | 64.7 | 50.0 | 77.1 | 45 |
| Male | White: Eastern European | 31-45 | No | 51 | 64.4 | 51.7 | 75.3 | 44 |
| Female | White: Any other White background | 18-30 | Yes | 32 | 63.5 | 50.8 | 74.5 | 43 |
| Female | White: Any other White background | 31-45 | No | 124 | 62.7 | 53.0 | 71.5 | 42 |
| Male | Jewish | 75+ | Yes | 31 | 62.6 | 47.9 | 75.3 | 41 |
| Male | White: Irish | 75+ | Yes | 3 | 62.3 | 43.0 | 78.4 | 40 |
| Female | White: Any other White background | 18-30 | No | 70 | 61.7 | 50.6 | 71.7 | 39 |
| Female | White: Any other White background | 46-59 | Yes | 50 | 61.4 | 49.5 | 72.1 | 38 |
| Male | Jewish | 60-74 | No | 1 | 60.8 | 44.4 | 75.0 | 37 |
| Male | White: Any other White background | 46-59 | No | 30 | 59.3 | 46.3 | 71.2 | 36 |
| Male | White: Irish | 60-74 | No | 3 | 59.2 | 41.0 | 75.2 | 35 |
| Female | White: Irish | 60-74 | No | 5 | 58.6 | 40.7 | 74.6 | 34 |
| Female | White: Irish | 75+ | Yes | 2 | 57.5 | 38.1 | 74.9 | 33 |
| Female | Jewish | 60-74 | No | 2 | 57.4 | 41.2 | 72.2 | 32 |
| Male | White: Any other White background | 18-30 | No | 26 | 56.8 | 43.6 | 69.0 | 31 |
| Male | White: Eastern European | 75+ | Yes | 1 | 55.8 | 37.7 | 72.5 | 30 |
| Female | White: Any other White background | 60-74 | Yes | 27 | 55.8 | 42.2 | 68.5 | 29 |
| Male | White: Eastern European | 60-74 | No | 2 | 55.4 | 38.6 | 71.0 | 28 |
| Female | White: Any other White background | 46-59 | No | 45 | 55.0 | 42.8 | 66.7 | 27 |
| Female | White: Eastern European | 60-74 | No | 2 | 53.1 | 36.6 | 69.0 | 26 |
| Male | White: Any other White background | 31-45 | No | 50 | 52.2 | 40.4 | 63.8 | 25 |
| Male | White: Any other White background | 60-74 | Yes | 21 | 50.7 | 37.0 | 64.3 | 24 |
| Female | White: Any other White background | 75+ | Yes | 3 | 48.0 | 31.7 | 64.7 | 23 |
| Male | White: Any other White background | 75+ | Yes | 3 | 44.4 | 28.6 | 61.3 | 22 |
| Female | White: Any other White background | 60-74 | No | 16 | 41.9 | 28.8 | 56.4 | 21 |
| Male | White: Any other White background | 60-74 | No | 8 | 41.1 | 27.4 | 56.2 | 20 |
| Male | White^ | 18-30 | Yes | 232 | 36.9 | 29.3 | 45.2 | 19 |
| Male | White | 31-45 | Yes | 480 | 33.8 | 27.5 | 40.9 | 18 |
| Male | White | 46-59 | Yes | 590 | 32.7 | 26.4 | 39.7 | 17 |
| Female | White: Any other White background | 75+ | No | 2 | 31.8 | 18.8 | 48.3 | 16 |
| Male | White: Any other White background | 75+ | No | 3 | 31.5 | 18.7 | 47.9 | 15 |
| Female | White | 31-45 | Yes | 660 | 28.7 | 23.1 | 35.0 | 14 |
| Male | White | 18-30 | No | 8 | 27.1 | 16.9 | 40.4 | 13 |
| Female | White | 18-30 | No | 7 | 25.8 | 16.0 | 39.0 | 12 |
| Female | White | 18-30 | Yes | 395 | 25.8 | 20.1 | 32.4 | 11 |
| Male | White | 60-74 | Yes | 655 | 25.6 | 20.0 | 32.2 | 10 |
| Female | White | 31-45 | No | 4 | 22.9 | 13.8 | 35.4 | 9 |
| Female | White | 46-59 | No | 2 | 22.4 | 13.3 | 35.1 | 8 |
| Male | White | 31-45 | No | 3 | 22.0 | 13.2 | 34.4 | 7 |
| Male | White | 46-59 | No | 5 | 21.6 | 12.9 | 34.0 | 6 |
| Female | White | 46-59 | Yes | 632 | 20.8 | 16.1 | 26.5 | 5 |
| Female | White | 60-74 | Yes | 427 | 15.7 | 11.5 | 21.2 | 4 |
| Male | White | 60-74 | No | 1 | 14.0 | 7.8 | 23.7 | 3 |
| Male | White | 75+ | Yes | 228 | 13.6 | 8.8 | 20.3 | 2 |
| Female | White | 75+ | Yes | 177 | 11.4 | 7.1 | 17.8 | 1 |

Table A.4. Full result, predicted percentage of experiencing lifetime racism, using 5-category ethnicity.

| sex | ethnicity (5 category) | age_cat | UK Nationality | n | Predicted % racism | low | high | Rank |
| --- | --- | --- | --- | --- | --- | --- | --- | --- |
| Female | Black, Black British, Black Welsh, Caribbean or African | 18-30 | Yes | 343 | 95.8 | 92.3 | 97.7 | 92 |
| Female | Black, Black British, Black Welsh, Caribbean or African | 31-45 | Yes | 309 | 94.0 | 89.5 | 96.6 | 91 |
| Male | Black, Black British, Black Welsh, Caribbean or African | 46-59 | Yes | 98 | 93.1 | 86.2 | 96.7 | 90 |
| Male | Black, Black British, Black Welsh, Caribbean or African | 18-30 | Yes | 275 | 92.6 | 87.3 | 95.8 | 89 |
| Female | Black, Black British, Black Welsh, Caribbean or African | 46-59 | Yes | 195 | 92.6 | 86.6 | 96.0 | 88 |
| Female | Asian, Asian British, Asian Welsh | 46-59 | Yes | 231 | 92.2 | 86.6 | 95.6 | 87 |
| Male | Black, Black British, Black Welsh, Caribbean or African | 31-45 | Yes | 185 | 91.3 | 84.8 | 95.2 | 86 |
| Male | Mixed or Multiple | 31-45 | Yes | 160 | 91.1 | 84.3 | 95.1 | 85 |
| Male | Asian, Asian British, Asian Welsh | 46-59 | Yes | 219 | 90.4 | 83.9 | 94.4 | 84 |
| Male | Other ethnic Group | 18-30 | Yes | 81 | 90.1 | 81.5 | 95.0 | 83 |
| Female | Black, Black British, Black Welsh, Caribbean or African | 60-74 | Yes | 44 | 90.1 | 79.3 | 95.6 | 82 |
| Female | Mixed or Multiple | 46-59 | Yes | 107 | 88.8 | 79.9 | 94.0 | 81 |
| Female | Black, Black British, Black Welsh, Caribbean or African | 46-59 | No | 16 | 88.3 | 74.1 | 95.3 | 80 |
| Female | Asian, Asian British, Asian Welsh | 18-30 | Yes | 760 | 88.2 | 82.4 | 92.2 | 79 |
| Male | Other ethnic Group | 31-45 | Yes | 99 | 88.1 | 79.1 | 93.6 | 78 |
| Female | Mixed or Multiple | 18-30 | Yes | 372 | 88.0 | 81.3 | 92.6 | 77 |
| Female | Mixed or Multiple | 31-45 | Yes | 239 | 87.9 | 80.5 | 92.7 | 76 |
| Male | Mixed or Multiple | 18-30 | No | 27 | 87.4 | 74.1 | 94.4 | 75 |
| Male | Asian, Asian British, Asian Welsh | 60-74 | Yes | 62 | 87.2 | 76.1 | 93.5 | 74 |
| Male | Mixed or Multiple | 46-59 | Yes | 55 | 86.7 | 75.1 | 93.4 | 73 |
| Female | Asian, Asian British, Asian Welsh | 31-45 | Yes | 668 | 86.7 | 80.3 | 91.2 | 72 |
| Male | Mixed or Multiple | 46-59 | No | 3 | 86.3 | 67.9 | 95.0 | 71 |
| Male | Asian, Asian British, Asian Welsh | 18-30 | Yes | 629 | 86.3 | 79.8 | 90.9 | 70 |
| Male | Asian, Asian British, Asian Welsh | 31-45 | Yes | 527 | 86.2 | 79.5 | 90.9 | 69 |
| Male | Mixed or Multiple | 18-30 | Yes | 225 | 85.9 | 77.6 | 91.4 | 68 |
| Male | Black, Black British, Black Welsh, Caribbean or African | 46-59 | No | 7 | 85.7 | 67.8 | 94.4 | 67 |
| Female | Mixed or Multiple | 60-74 | Yes | 27 | 85.4 | 70.8 | 93.4 | 66 |
| Female | Other ethnic Group | 18-30 | Yes | 131 | 85.3 | 76.0 | 91.4 | 65 |
| Female | Mixed or Multiple | 31-45 | No | 57 | 84.3 | 72.0 | 91.8 | 64 |
| Female | Black, Black British, Black Welsh, Caribbean or African | 18-30 | No | 64 | 84.1 | 72.1 | 91.5 | 63 |
| Male | Black, Black British, Black Welsh, Caribbean or African | 18-30 | No | 49 | 83.6 | 70.4 | 91.6 | 62 |
| Female | Other ethnic Group | 46-59 | No | 13 | 83.4 | 65.8 | 92.9 | 61 |
| Female | Other ethnic Group | 31-45 | Yes | 128 | 83.4 | 73.4 | 90.1 | 60 |
| Female | Black, Black British, Black Welsh, Caribbean or African | 60-74 | No | 2 | 83.3 | 61.9 | 93.8 | 59 |
| Male | Black, Black British, Black Welsh, Caribbean or African | 60-74 | No | 1 | 82.7 | 60.5 | 93.7 | 58 |
| Female | Mixed or Multiple | 18-30 | No | 57 | 82.5 | 69.6 | 90.7 | 57 |
| Male | Black, Black British, Black Welsh, Caribbean or African | 60-74 | Yes | 22 | 82.3 | 65.5 | 91.9 | 56 |
| Male | Other ethnic Group | 46-59 | Yes | 71 | 82.2 | 69.9 | 90.1 | 55 |
| Female | Asian, Asian British, Asian Welsh | 18-30 | No | 198 | 82.1 | 72.9 | 88.7 | 54 |
| Female | Black, Black British, Black Welsh, Caribbean or African | 31-45 | No | 86 | 81.4 | 69.5 | 89.3 | 53 |
| Male | Mixed or Multiple | 60-74 | Yes | 20 | 81.1 | 63.5 | 91.4 | 52 |
| Female | Asian, Asian British, Asian Welsh | 46-59 | No | 34 | 81.1 | 65.9 | 90.5 | 51 |
| Male | Asian, Asian British, Asian Welsh | 46-59 | No | 21 | 80.0 | 62.7 | 90.5 | 50 |
| Female | Asian, Asian British, Asian Welsh | 60-74 | Yes | 45 | 79.9 | 65.2 | 89.4 | 49 |
| Male | Asian, Asian British, Asian Welsh | 18-30 | No | 150 | 79.5 | 69.0 | 87.1 | 48 |
| Female | Mixed or Multiple | 46-59 | No | 7 | 79.0 | 57.7 | 91.2 | 47 |
| Male | Mixed or Multiple | 60-74 | No | 2 | 78.9 | 55.0 | 92.0 | 46 |
| Female | Mixed or Multiple | 60-74 | No | 5 | 77.6 | 54.4 | 90.9 | 45 |
| Female | Other ethnic Group | 46-59 | Yes | 117 | 76.6 | 64.5 | 85.5 | 44 |
| Male | Mixed or Multiple | 31-45 | No | 26 | 76.4 | 58.8 | 88.0 | 43 |
| Female | Black, Black British, Black Welsh, Caribbean or African | 75+ | Yes | 1 | 76.0 | 49.4 | 91.1 | 42 |
| Male | Other ethnic Group | 46-59 | No | 6 | 75.9 | 53.2 | 89.7 | 41 |
| Female | Asian, Asian British, Asian Welsh | 31-45 | No | 169 | 75.5 | 64.4 | 83.9 | 40 |
| Female | Other ethnic Group | 18-30 | No | 49 | 75.4 | 60.3 | 86.1 | 39 |
| Male | Black, Black British, Black Welsh, Caribbean or African | 31-45 | No | 59 | 75.1 | 60.5 | 85.6 | 38 |
| Female | Other ethnic Group | 31-45 | No | 46 | 75.0 | 59.6 | 85.9 | 37 |
| Male | Other ethnic Group | 60-74 | Yes | 109 | 72.8 | 59.4 | 83.1 | 36 |
| Male | Other ethnic Group | 18-30 | No | 20 | 72.4 | 53.2 | 85.9 | 35 |
| Male | Other ethnic Group | 31-45 | No | 25 | 72.1 | 53.7 | 85.2 | 34 |
| Male | Asian, Asian British, Asian Welsh | 60-74 | No | 2 | 71.8 | 46.0 | 88.4 | 33 |
| Male | Asian, Asian British, Asian Welsh | 75+ | Yes | 13 | 70.5 | 47.4 | 86.3 | 32 |
| Male | Asian, Asian British, Asian Welsh | 31-45 | No | 96 | 70.3 | 57.0 | 80.8 | 31 |
| Female | Mixed or Multiple | 75+ | Yes | 1 | 70.2 | 42.2 | 88.4 | 30 |
| Male | Other ethnic Group | 60-74 | No | 1 | 69.9 | 43.1 | 87.6 | 29 |
| Male | Mixed or Multiple | 75+ | Yes | 3 | 68.4 | 41.1 | 87.0 | 28 |
| Female | White | 31-45 | No | 249 | 68.3 | 57.5 | 77.5 | 27 |
| Female | White | 18-30 | No | 152 | 67.1 | 55.0 | 77.2 | 26 |
| Female | Asian, Asian British, Asian Welsh | 60-74 | No | 2 | 66.5 | 40.1 | 85.5 | 25 |
| Male | White | 18-30 | No | 70 | 64.7 | 50.2 | 76.9 | 24 |
| Female | Other ethnic Group | 75+ | Yes | 28 | 64.6 | 44.0 | 80.9 | 23 |
| Female | Mixed or Multiple | 75+ | No | 1 | 64.4 | 35.2 | 85.8 | 22 |
| Female | Other ethnic Group | 60-74 | Yes | 125 | 63.2 | 49.2 | 75.2 | 21 |
| Male | Other ethnic Group | 75+ | Yes | 34 | 62.8 | 42.9 | 79.2 | 20 |
| Female | Asian, Asian British, Asian Welsh | 75+ | Yes | 7 | 62.7 | 37.4 | 82.6 | 19 |
| Female | White | 46-59 | No | 81 | 61.2 | 46.7 | 74.0 | 18 |
| Female | Other ethnic Group | 60-74 | No | 4 | 59.5 | 34.1 | 80.7 | 17 |
| Male | White | 46-59 | No | 57 | 59.4 | 43.7 | 73.5 | 16 |
| Male | White | 31-45 | No | 116 | 56.1 | 43.0 | 68.3 | 15 |
| Male | White | 60-74 | No | 14 | 47.5 | 27.4 | 68.4 | 14 |
| Male | White | 18-30 | Yes | 276 | 45.0 | 34.0 | 56.5 | 13 |
| Female | White | 60-74 | No | 23 | 44.0 | 26.2 | 63.5 | 12 |
| Male | White | 31-45 | Yes | 609 | 43.8 | 33.7 | 54.5 | 11 |
| Female | White | 31-45 | Yes | 834 | 39.6 | 30.0 | 50.1 | 10 |
| Male | White | 46-59 | Yes | 666 | 37.2 | 27.8 | 47.8 | 9 |
| Female | White | 18-30 | Yes | 488 | 32.8 | 23.8 | 43.2 | 8 |
| Male | White | 60-74 | Yes | 705 | 29.1 | 20.6 | 39.4 | 7 |
| Female | White | 46-59 | Yes | 714 | 25.4 | 18.0 | 34.5 | 6 |
| Female | White | 60-74 | Yes | 488 | 22.1 | 14.9 | 31.4 | 5 |
| Female | White | 75+ | No | 2 | 21.6 | 8.1 | 46.4 | 4 |
| Male | White | 75+ | No | 3 | 20.8 | 7.8 | 44.8 | 3 |
| Male | White | 75+ | Yes | 240 | 16.8 | 9.7 | 27.5 | 2 |
| Female | White | 75+ | Yes | 186 | 14.0 | 7.7 | 24.2 | 1 |

Supplementary Table 5. Parameter estimates for logistic models of experience of racism in lifetime, null intersectional model (a) vs additive main effect model (b), comparing model using social strata constructed from 21-category ethnicity (left) and 5-category ethnicity (right), using Markov chain Monte Carlo simulations with 5000 iterations and 500 burnin; using runmlwin package on Stata (Leckie & Charlton, 2013)

|  |  | Logistic Model 1A | | Logistic Model 1B | | |  |  | Logistic Model 2A | | Logistic Model 2B | | |
| --- | --- | --- | --- | --- | --- | --- | --- | --- | --- | --- | --- | --- | --- |
|  |  | OR | 95% CI | OR | | 95% CI |  |  | OR | 95% CI | OR | | 95% CI |
| Fixed Effects: Regression Coefficients | | |  |  | |  |  |  |  |  |  |  | |
| Intercept |  | 4.29 | [3.74-4.88] | 0.5 | | [0.38-0.62] | Intercept |  | 3.46 | [2.81-4.37] | 1.19 | [0.76-1.70] | |
| Sex |  |  |  |  | |  | Sex |  |  |  |  |  | |
|  | Female (Ref) |  |  | 1 | | [-] |  | Female (Ref) | |  | 1 | [-] | |
|  | Male |  |  | 1.01 | | [0.86-1.18] |  | Male |  |  | 1.01 | [0.69-1.46] | |
| Ethnicity |  |  |  |  | |  | Ethnicity |  |  |  |  |  | |
|  | Asian: Bangladeshi | |  | 15.95 | | [9.95-25.51] |  | Asian |  |  | 5.99 | [4.05-8.58] | |
|  | Asian: Chinese |  |  | 18.52 | | [12.32-27.43] |  | Black |  |  | 9.74 | [6.11-14.83] | |
|  | Asian: Indian |  |  | 13.15 | | [9.04-18.26] |  | Mixed |  |  | 7.32 | [4.61-11.14] | |
|  | Asian: Pakistani |  |  | 14.74 | | [10.24-21.35] |  | White (Ref) | |  | 1 | [-] | |
|  | Asian: Any other Asian background | | | 16.31 | | [11.38-22.85] |  | Other |  |  | 4.49 | [2.92-6.46] | |
|  | Black: African |  |  | 20.27 | | [14.03-29.22] |  |  |  |  |  |  | |
|  | Black: Caribbean | |  | 43.24 | | [26.19-70.28] |  |  |  |  |  |  | |
|  | Black: Any other Black/African/Caribbean background | | | 33.79 | | [15.97-64.64] |  |  |  |  |  |  | |
|  | Mixed: White and Asian | |  | 15.01 | | [9.79-21.92] |  |  |  |  |  |  | |
|  | Mixed: White and Black African | | | 33.93 | | [16.3-68.44] |  |  |  |  |  |  | |
|  | Mixed: White and Black Caribbean | | | 20.88 | | [12.31-32.91] |  |  |  |  |  |  | |
|  | Mixed: Any other mixed/multiple background | | | 14.27 | | [9.14-21.8] |  |  |  |  |  |  | |
|  | White: Eastern European | |  | 7.5 | | [4.9-11.08] |  |  |  |  |  |  | |
|  | White: English / Welsh / Scottish / Northern Ireland/ British (Ref) | | | 1 | | [-] |  |  |  |  |  |  | |
|  | White: Gypsy/Traveller | |  | 19.3 | | [11.3-30.55] |  |  |  |  |  |  | |
|  | White: Irish |  |  | 8.79 | | [5.17-13.98] |  |  |  |  |  |  | |
|  | White: Roma |  |  | 9.23 | | [4.8-16.27] |  |  |  |  |  |  | |
|  | White: Any other White background | | | 4.66 | | [3.36-6.51] |  |  |  |  |  |  | |
|  | Other: Arab |  |  | 9.78 | | [5.72-15.52] |  |  |  |  |  |  | |
|  | Other: Any other ethnic group | |  | 14.06 | | [9.27-21] |  |  |  |  |  |  | |
|  | Jewish |  |  | 9.99 | | [6.99-14.2] |  |  |  |  |  |  | |
| Age Category |  |  |  |  | |  | Age Category |  |  |  |  |  | |
|  | 18 - 30 years (Ref) | |  | 1 | | [-] |  | 18 - 30 years (Ref) | |  | 1 | [-] | |
|  | 31 - 45 years |  |  | 0.93 | | [0.79-1.11] |  | 31 - 45 years | |  | 0.85 | [0.59-1.19] | |
|  | 46 - 59 years |  |  | 0.96 | | [0.76-1.11] |  | 46 - 59 years | |  | 0.89 | [0.63-1.34] | |
|  | 60 - 74 years |  |  | 0.57 | | [0.43-0.75] |  | 60 - 74 years | |  | 0.57 | [0.32-0.85] | |
|  | 75+ years |  |  | 0.37 | | [0.23-0.53] |  | 75+ years | |  | 0.3 | [0.15-0.53] | |
| UK Citizen |  |  |  |  | |  | UK Citizen |  |  |  |  |  | |
|  | Yes (Ref) |  |  | 1 | | [-] |  | Yes (Ref) | |  | 1 | [-] | |
|  | No |  |  | 0.56 | | [0.46-0.67] |  | No |  |  | 0.78 | [0.57-1.08] | |
|  |  |  |  |  | |  |  |  |  |  |  |  | |
| Random Effects: Variances | |  |  |  | |  | Random Effects: Variances | | |  |  |  | |
| Stratum-level |  | 1.16 | [0.91-1.47] | 0.12 | | [0.06-0.20] | Stratum-level |  | 1.22 | [0.87-1.70] | 0.33 | [0.22-0.50] | |
|  |  |  |  | |  |  |  |  |  |  |  |  | |
| Summary Statistics | |  |  |  | |  | Summary Statistics | |  |  |  |  | |
| Variance Partition Coefficient (VPC) | | 26.09% |  | 3.66% | |  | Variance Partition Coefficient (VPC) | | 27.09% |  | 9.19% |  | |
| Proportional Change in Variance (PCV) | | |  | 89.20% | |  | Proportional Change in Variance (PCV) | | |  | 72.80% |  | |
